# Application value of Xpert Carba-R combined with LAMP in the diagnosis and prognosis of carbapenem-resistant Gram-negative bacilli pneumonia

**DOI:** 10.1128/spectrum.02130-25

**Published:** 2026-01-09

**Authors:** Jiannan Hu, Xinyue Ma, Jingjing Sun, Xinghui Gao, Yi-Wei Tang, Chao Liu, Decai Wang, Yunfei Zhu, Minrong Liu, Shuyun Xu

**Affiliations:** 1 Department of Respiratory and Critical Care Medicine, Key Laboratory of Pulmonary Diseases of the Health Ministry, Tongji Hospital, Tongji Medical College, Huazhong University of Science and Technology 12443, Wuhan, Hubei, China; 2 Medical Affairs, Danaher/Cepheid, Shanghai, China; MultiCare Health System, Tacoma, Washington, USA

**Keywords:** Xpert Carba-R, LAMP, carbapenem resistance, pneumonia, early diagnosis, early treatment

## Abstract

Given the current global challenges posed by antimicrobial resistance, the early and accurate identification of carbapenem-resistant Gram-negative bacilli (CR-GNB) is crucial for patient outcomes. This study evaluates the combined diagnostic performance of Xpert Carba-R and Loop-mediated isothermal amplification (LAMP) in the early detection of CR-GNB in patients with hospital-acquired pneumonia (HAP) or ventilator-associated pneumonia (VAP) and investigates the effect of early treatment based on detection results on the prognosis of these patients. In this single-center prospective study, 250 HAP/VAP patients were enrolled. Respiratory specimens underwent bacterial culture, antimicrobial susceptibility testing, Xpert Carba-R, and LAMP. Diagnostic performance (sensitivity, specificity, positive predictive value [PPV], negative predictive value [NPV]) of the combined test to detect CR-GNB was assessed. Clinical utility in guiding early antibiotic therapy was evaluated. The combined method demonstrated 64.87% sensitivity (95% CI: 52.89%–75.61%) and 94.32% specificity (95% CI: 89.80%–97.24%), with perfect 100% sensitivity and specificity for detecting CR-GNB in *Klebsiella pneumoniae*. Patients testing positive were randomized to early or non-early treatment groups. For severe patients, the early treatment group showed significantly improved clinical outcomes, including lower acute physiology and chronic health evaluation-II (APACHE-II) scores (*P* < 0.05) and sequential organ failure assessment (SOFA) scores (*P* < 0.01) at 15 days post-treatment, along with reduced 30-day hospitalization costs (*P* < 0.05). Notably, among the critically ill patients, compared with the non-early treatment group, the early treatment group achieved a clinical improvement rate of 81.25% and a microbiological eradication rate of 56.25%, showing a clear advantage.

**IMPORTANCE:**

An urgent clinical need exists for rapid detection of carbapenem-resistant Gram-negative bacilli (CR-GNB) in respiratory specimens to facilitate early and precise antibiotic therapy. To address this gap, we developed an innovative dual-method assay: Xpert Carba-R to detect five major carbapenemases, and loop-mediated isothermal amplification (LAMP) to identify common Gram-negative respiratory pathogens. Integration of these methods enables simultaneous and early determination of bacterial species and their resistance profiles. We validated this assay against conventional culture-based antimicrobial susceptibility testing (AST) and assessed its clinical impact on patient outcomes. Results indicate that this integrated approach provides clinically actionable data, serving as a viable alternative to conventional diagnostic workflows.

This study is registered with the Chinese Clinical Trial Registry as ChiCTR2400090694.

## INTRODUCTION

Hospital-acquired pneumonia (HAP) and ventilator-associated pneumonia (VAP) are severe infections associated with high mortality rates. Multidrug-resistant Gram-negative bacilli are common pathogens in HAP/VAP (1), and carbapenems are typically used to treat these infections. However, the rise in carbapenem-resistant Gram-negative bacilli (CR-GNB) infections is alarming, with resistance rates reaching 12% in HAP/VAP (2). CR-GNB infections pose a significant health threat and increase the socioeconomic burden, requiring urgent prevention and control measures.

Current diagnostic practices rely on conventional bacterial culture and antibiotic susceptibility test (AST), which are time-consuming (taking over 3 days) and inadequate for timely intervention. Furthermore, empirical antibiotic therapy often starts before culture results are available, which may decrease detection rates of CR-GNB, increasing treatment failure rates and costs. Therefore, rapid detection methods for CR-GNB are urgently needed to guide early precision treatment.

Carbapenemase production is the primary mechanism behind carbapenem resistance in CR-GNB, typically classified into three classes based on the Ambler classification system. Class A comprises serine carbapenemases such as *Klebsiella pneumoniae* carbapenemase (KPC) type, Class B encompasses metallo-β-lactamases like New Delhi metallo-β-lactamase (NDM), Verona integron encoded metallo-β-lactamase (VIM), and imipenemase metallo-β-lactamase (IMP); Class D comprises oxacillinase enzyme family, such as Oxacillinase 48 type carbapenemases (OXA-48) (3). Therefore, identifying specific carbapenemase enzyme types is instrumental in guiding antibiotic selection for CR-GNB infections (4). Antibiotic options for CR-GNB infections exhibit variable antibacterial activity against distinct carbapenemase types: for instance, ceftazidime/avibactam is ineffective against the NDM type (5). Additionally, bacterial species represent another crucial factor influencing antibiotic selection following CR-GNB infections (6): *Pseudomonas aeruginosa* naturally exhibits resistance to tigecycline (7), thus tigecycline is commonly used to treat carbapenem-resistant enterobacteriaceae and carbapenem-resistant *Acinetobacter baumannii* (CRAB) infections, but cannot be utilized for treating carbapenem-resistant *Pseudomonas aeruginosa* (CRPA) infections; furthermore, avibactam combinations are ineffective against the majority of CRAB strains.

Xpert Carba-R (Cepheid, Sunnydale, CA) is a fluorescence-based quantitative polymerase chain reaction (PCR) assay designed to detect carbapenemase genotypes, which can rapidly detect five of the most common carbapenemase genotypes, including KPC, NDM, VIM, IMP, and OXA-48 within 1 h (8). Besides, Xpert Carba-R has demonstrated high predictive performance for detecting carbapenemase genes in intestinal microbiota, with reported sensitivity and specificity ranging from 96.6% to 100% and 94.2% to 100%, respectively (9). However, there are limited studies on the direct detection of respiratory samples using Xpert Carba-R. One study attempted to directly detect carbapenemase genes in sputum specimens, and the results showed a high concordance rate for the detection of resistance genes compared to PCR identification of these genes in bacterial colonies after culture (10). Therefore, propose integrating loop-mediated isothermal amplification (LAMP) to identify bacterial species. LAMP allows rapid detection of 13 common pathogens, including Gram-negative bacilli associated with HAP/VAP, with advantages such as speed (around 2 h), simplicity, and high sensitivity and specificity (11, 12).

Based on this, we hypothesize a combination of Xpert Carba-R and LAMP to enable rapid detection of CR-GNB, leading to early precision therapy that ultimately benefits patients. This study will evaluate the diagnostic value of this combined assay and its effect on the prognosis of CR-GNB pneumonia patients.

## MATERIALS AND METHODS

### Study design and participants

In this prospective study, inclusion criteria were as follows: (i) clinical diagnosis of pneumonia based on the ATS/IDSA Clinical Practice Guideline (13) and Chinese Guidelines (14) for HAP/VAP; (ii) age ≥18 years; (iii) consent to induced sputum collection or bronchoscopy with bronchoalveolar lavage. The exclusion criteria were as follows: (i) lower respiratory tract imaging changes caused by non-infectious diseases, including pulmonary edema, malignant tumors of the lungs, and heart failure. (ii) Missing or incomplete clinical data, or incomplete or aborted examination. (iii) Non-pulmonary infection and non-Gram-negative bacterial infection.

**Fig 1:**
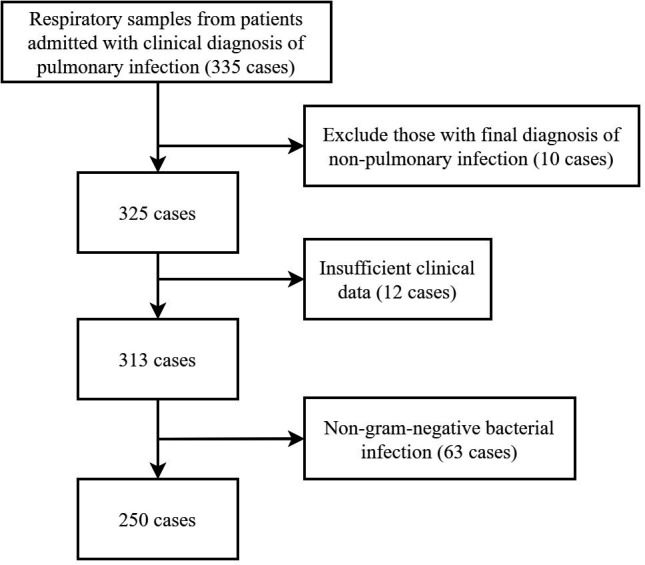
Flowchart of patient screening.

A total of 335 patients were initially enrolled based on the inclusion criteria. After excluding 10 patients with non-pulmonary infections, 12 with incomplete microbiological testing, and 63 with non-Gram-negative bacterial infections, 250 patients remained for analysis (Fig. 1). Data on patient characteristics, medical history, laboratory results, radiological findings, microbiology results (Xpert Carba-R, LAMP, bacterial culture, and AST), treatment regimens, medical costs, and outcomes were collected from electronic medical records and analyzed. In this study, patients with positive combined detection results were assigned to either the early treatment group or the non-early treatment group using simple randomization. The clinicians in the early treatment group were informed of the rapid test results and implemented precise antibiotic therapy within 48 h. The clinicians in the non-early treatment group were not informed of the rapid test results and adjusted treatment solely based on clinical experience and subsequent bacterial culture and AST results. However, all outcome assessments and data analyses were conducted under blinded conditions. We will evaluate the prognosis of the two groups by analyzing the following indicators: all-cause mortality at 28 and 90 days, changes in acute physiology and chronic health evaluation-II (APACHE II) score and sequential organ failure assessment (SOFA) score for 15 days after the start of antibiotic treatment, clinical improvement (compared to the baseline, at least one of the following showed significant improvement in the patient’s symptoms and signs, laboratory inflammatory markers, or chest computed tomography lesions), readmission rate, microbial clearance rate, oxygenation improvement, the time to normalize temperature and white blood cell count, mechanical ventilation time within 30 days, ICU stay within 30 days, antibiotic cost within 15 days, total hospitalization cost within 30 days, etc. We will screen the critically ill patients in the two groups for focused analysis based on the criteria of APACHE II score ≥15 (15) or SOFA score ≥4 (16).

### Xpert Carba-R

The Xpert Carba-R kits used in this study were provided free of charge by Cepheid. The sputum/bronchoalveolar lavage fluid (BALF) was preprocessed before entering the Xpert cartridge. Specimens are mixed with liquefaction reagent (4% NaOH) at a volumetric ratio of 1:2, vigorously shaken, and then incubated at 37°C. After the specimen was fully liquefied, an equal volume of sample reagent was added, and 1.7 mL of the mixture was transferred into the cartridge for testing. The results were automatically displayed by the GeneXpert system within 53 min.

### Loop-mediated isothermal amplification

The bacterial genomic DNA extraction kit and the Crystal Respiratory Pathogen Nucleic Acid Detection (LAMP) kit used for respiratory sample testing were both sourced from BGI Group Limited. Nucleic acid extraction from lower respiratory tract secretions, preparation of a constant temperature amplification reaction system, chip sampling and centrifugation, nucleic acid amplification, and result interpretation were carried out in accordance with the instructions.

### Statistical analysis

Categorical variables will be presented as frequencies and percentages. Normality tests will be performed on continuous variables, and those with a normal distribution will be expressed as means with standard deviations; non-normally distributed variables will be reported as medians with interquartile ranges. Group differences will be assessed using *t*-tests for normally distributed continuous variables and Mann-Whitney *U* tests for non-normally distributed variables. For categorical variables, two-sample tests or Fisher’s exact tests will be used for comparisons. Diagnostic performance will be evaluated using the area under the curve (AUC). Sensitivity, specificity, positive predictive value (PPV), and negative predictive value (NPV) will be calculated using MedCalc software. All *P* values will be two-tailed, with statistical significance set at *P* < 0.05. Statistical analysis will be conducted using IBM SPSS 26.0, MedCalc software, and GraphPad Prism 9.

## RESULTS

### Clinical characteristics of patients

This study included 250 patients, with a mean age of 61.88 years (range: 18 to 89 years). The majority of participants were male (*n* = 164, 65.60%). A total of 229 patients were hospitalized in the respiratory ward, 130 (52.00%) were in the respiratory intensive care unit, and 99 (39.60%) were in the general respiratory ward. Geographically, 91.60% of the patients were from Hubei Province, China. Common comorbidities included hypertension (36.40%), chronic obstructive pulmonary disease (COPD) (16.4%), tumors (23.20%), and diabetes (10.2%). Among the patients, 118 (47.20%) had ventilator-associated pneumonia. Additionally, 29.20% had a history of smoking, 37.20% had undergone surgery in the past 6 months, 57.20% had respiratory failure, 56.40% had hypoproteinemia, and 70.40% had recently used restrictive antibiotics. Laboratory results showed elevated levels of procalcitonin (PCT) and high-sensitivity C-reactive protein (hsCRP) in most patients, and 49.60% had decreased lymphocyte counts. A total of 250 respiratory specimens were collected, consisting of 68 sputum samples (28.57%) and 182 BALF samples (72.80%). The median time from infection onset to hospital admission was 10 days, with an interquartile range (IQR) of 5 to 19 days. Based on respiratory sample bacterial culture results, 9.60% of patients had multiple isolates from the same patient (Table 1).

**TABLE 1:** Demographic and clinical characteristics of included patients^*d*^

Patient characteristics	Gram-negative bacterium pneumonia (*N* = 250)
Age (years), mean (range)	61.88 (18–89)
Sex	
Female, *n* (%)	86 (34.40%)
Male, *n* (%)	164 (65.60%)
Location of care	
General respiratory ward, *n* (%)	99 (39.60%)
Respiratory intensive care unit, *n* (%)	130 (52.00%)
Other wards, *n* (%)	21 (8.40%)
Geographical origin	
Hubei Province, China, *n* (%)	229 (91.60%)
Other provinces, China, *n* (%)	21 (8.40%)
Smoking history, *n* (%)	73 (29.20%)
Past medical history	
Coronary heart disease, *n* (%)	32 (12.80%)
Stroke, *n* (%)	40 (16.00%)
Diabetes, *n* (%)	48 (19.20%)
Hypertension, *n* (%)	91 (36.40%)
Tuberculosis, *n* (%)	22 (8.80%)
COPD, *n* (%)	38 (15.20%)
Bronchiectasis, *n* (%)	30 (12.00%)
Tumors, *n* (%)	58 (23.20%)
Use of immunosuppressants, *n* (%)	21 (8.40%)
History of surgical procedures, *n* (%)	118 (47.20%)
Use of invasive mechanical ventilation, *n* (%)	118 (47.20%)
Use of restricted-use antibiotics, *n* (%)	176 (70.40%)
Respiratory failure, *n* (%)	143 (57.20%)
Laboratory findings	
Decreased lymphocyte count^*a*^, *n* (%)	124 (49.60%)
Elevated PCT^*b*^, *n* (%)	157 (62.80%)
Elevated hsCRP^*c*^, *n* (%)	212 (84.80%)
Hypoalbuminemia, *n* (%)	141 (56.40%)
Isolation site	
Respiratory tract, *n* (%)	250 (100.00%)
Sample type	
Sputum, *n* (%)	74 (29.60%)
Bronchoalveolar lavage fluid, *n* (%)	176 (70.40%)
Time of infection onset relative to hospital admission, median (IQR)	10 (5–19)
Multiple isolates came from the same patient, *n* (%)	24 (9.60%)

^*a*^ Lymphocyte count < 1.10 × 10^9^/L.

^*b*^ PCT > 0.05 ng/mL.

^*c*^ hsCRP > 10 mg/L.

^*d*^ COPD, chronic obstructive pulmonary disease; PCT, procalcitonin; hsCRP, high-sensitivity C-reactive protein; IQR, interquartile range.

### The diagnostic accuracy of Xpert Carba-R combined with LAMP

A total of 250 respiratory specimens were collected in this study, of which 74 (29.60%) sputum/BALF specimens were CR-GNB positive and 176 (70.40%) were negative. Of the 67 sputum samples, 15 (22.39%) were positive for CR-GNB. Of 183 samples of BALF, 59 (32.24%) were CR-GNB positive. A total of 35.14% (26/74) of the samples were positive for carbapenem resistance, while the Xpert Carba-R combined LAMP test was negative. (Table 2).

**TABLE 2:** Distribution of Xpert Carba-R combined LAMP and culture drug sensitivity detection results^*c*^

Xpert Carba-R combined with LAMP	Bacterial culture and drug sensitivity test		Total
	CR-GNB	Non-CR-GNB	
Positive^*a*^	48	10	58
Negative^*b*^	26	166	192
Total	74	176	250

^*a*^ Positive combined test result was defined as the detection of a carbapenemase genotype by Xpert Carba-R and the detection of Gram-negative bacilli by LAMP.

^*b*^ Negative combined test result was defined as either: (i) no detection by both Xpert Carba-R and LAMP, or (ii) no detection by either one of the two tests.

^*c*^ CR-GNB, carbapenem-resistant Gram-negative bacilli.

**Fig 2:**
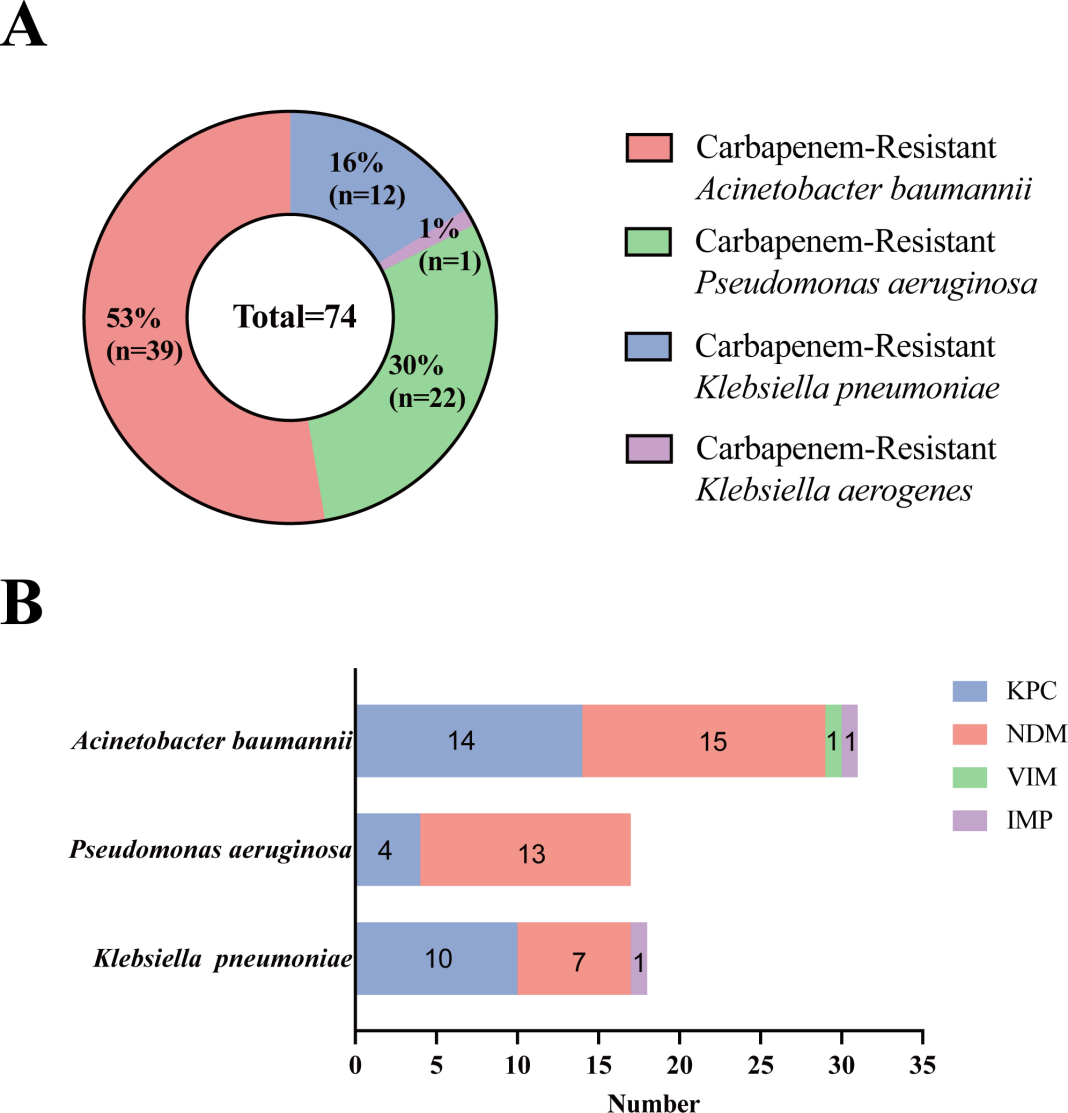
Distribution of CR-GNB in respiratory tract samples (**A**) and distribution of carbapenemase genotypes in different bacterial species (**B**).

Based on the bacterial culture results of respiratory specimens, the following GNB were mainly detected in respiratory specimens (sputum/BALF): *Pseudomonas aeruginosa* (75 cases), *Acinetobacter baumannii* (45 cases), and *Klebsiella pneumoniae* (26 cases). Among them, 74 cases of CR-GNB were detected: *Pseudomonas aeruginosa* (22 cases, 29.73%), *Acinetobacter baumannii* (39 cases, 52.70%), *Klebsiella aerogenes* (1 case, 1.35%), and *Klebsiella pneumoniae* (12 cases, 16.22%) (Fig. 2A).

Our study shows that different bacterial strains can carry multiple carbapenemase genotypes. In *Acinetobacter baumannii* isolates, we detected the following carbapenemase genotypes: 14 KPC, 15 NDM, 1 VIM, 1 IMP; in *Pseudomonas aeruginosa* isolates, we detected the following carbapenemase genotypes: 4 KPC, 13 NDM; in *Klebsiella pneumoniae* isolates, we detected the following carbapenemase genotypes: 10 KPC, 7 NDM, 1 IMP. Thus, in our data, KPC and NDM are the two carbapenemase genotypes that are more common in GNB. Compared with KPC and NDM, IMP, VIM, and OXA-48 are relatively rare genotypes, and OXA-48 has not appeared in our detection (Fig. 2B).

The sensitivity, specificity, PPV, and NPV of Xpert Carba-R combined with LAMP in the diagnosis of CR-GNB pneumonia are shown in Table 3. All respiratory specimens were examined, and the sensitivity, specificity, PPV, and NPV were 64.87% (95% CI 52.89–75.61), 94.32% (95% CI 89.80–97.24), 82.76% (95% CI 71.99–89.97), and 86.46% (95% CI 82.38–89.71), respectively. The sensitivity of combined detection in BALF was slightly better than that in sputum. In addition, we comprehensively evaluated the diagnostic efficiency of Xpert Carba-R combined LAMP detection by calculating the AUC. The AUC of combined detection (sputum/BALF) was 0.796. For BALF and sputum samples, the AUCs were 0.742 and 0.814, respectively. This suggests that the combined detection has certain diagnostic value.

**TABLE 3:** Diagnostic accuracy of Xpert Carba-R combined with LAMP in detecting CR-GNB in different respiratory specimens^*a*^

Sample types	Sensitivity (%) 95% CI	Specificity (%) 95% CI	PPV (%) 95% CI	NPV (%) 95% CI	AUC
Sputum/BALF	64.87 (52.89–75.61)	94.32 (89.80–97.24)	82.76 (71.99–89.97)	86.46 (82.38–89.71)	0.796
Sputum	60.00 (32.29–83.66)	88.46 (76.56–95.65)	60.00 (38.86–77.97)	88.46 (80.37–93.49)	0.742
BALF	66.10 (52.61–77.92)	96.77 (91.95–99.11)	90.70 (78.52–96.30)	85.71 (80.75–89.56)	0.814

^*a*^ PPV, positive predictive value; NPV, negative predictive value; CI, confidence interval; BALF, bronchoalveolar lavage fluid; AUC, area under the curve.

In order to evaluate the ability of Xpert Carba-R combined with LAMP to detect carbapenemases in different strains, we statistically analyzed the sensitivity, specificity, PPV, and NPV of the combined assay for the three main GNB (*Klebsiella pneumoniae*, *Pseudomonas aeruginosa,* and *Acinetobacter baumannii*) detected by this combined assay. Surprisingly, the combined assay had excellent ability to detect carbapenemase of *Klebsiella pneumoniae*. The results showed that for *Klebsiella pneumoniae*, the sensitivity, specificity, PPV, and NPV were 100.00% (95% CI 73.54–100.00), 100.00% (95% CI 75.30–100.00), 100.00% (95% CI 73.54–100.00), and 100.00% (95% CI 75.30–100.00), respectively. The sensitivity of the combined assay in detecting *Pseudomonas aeruginosa* carbapenemase was only 54.55%. For *Acinetobacter baumannii*, the sensitivity was 64.51% (Table 4).

**TABLE 4:** Diagnostic accuracy of Xpert Carba-R combined with LAMP in detecting CR-GNB in different bacterial strains^*a*^

Strains	Sensitivity (%) 95% CI	Specificity (%) 95% CI	PPV (%) 95% CI	NPV (%) 95% CI	AUC
*Klebsiella pneumoniae*	100.00 (73.54–100.00)	100.00 (75.30–100.00)	100.00 (73.54–100.00)	100.00 (75.30–100.00)	1.00
*Pseudomonas aeruginosa*	54.55 (32.21–75.61)	94.00 (83.45–98.75)	80.00 (55.60–92.74)	82.46 (74.73–88.19)	0.74
*Acinetobacter baumannii*	61.54 (44.62–76.64)	100.00 (47.82–100.00)	100.00 (85.75–100.00)	25.00 (18.31–33.15)	0.81

^*a*^ PPV, positive predictive value; NPV, negative predictive value; CI, confidence interval; BALF, bronchoalveolar lavage fluid; AUC, area under the curve.

In addition, we comprehensively analyzed the diagnostic efficiency of Xpert Carba-R combined LAMP detection in detecting CR-GNB in different strains by calculating the AUC. The results showed that Xpert Carba-R combined with LAMP had high diagnostic efficiency in detecting carbapenemase resistance gene in *Klebsiella pneumoniae*, with an AUC of 1.000. For *Pseudomonas aeruginosa* and *Acinetobacter baumannii*, the AUCs were 0.743 and 0.808, respectively.

### The effect of early treatment according to the early detection results of Xpert Carba-R combined with LAMP on the prognosis of patients

In order to explore the impact of the combined test on the prognosis of patients, we randomly divided the patients with positive combined test results into an early treatment group and a non-early treatment group. The early treatment group received precise antibiotic therapy within 48 h based on the Xpert Carba-R combined with the LAMP test, while the non-early treatment group received empirical antibiotics until bacterial culture and drug sensitivity were detected (3–5 days after sample submission). We assessed the time from hospital admission to the start of appropriate treatment in both groups. The early treatment group took (2.875 ± 1.147) days, while the non-early treatment group took (5.357 ± 1.393) days. The delay in starting appropriate antibiotics was, on average, 2.5 days longer in the non-early treatment group due to the lack of early detection.

**Fig 3:**
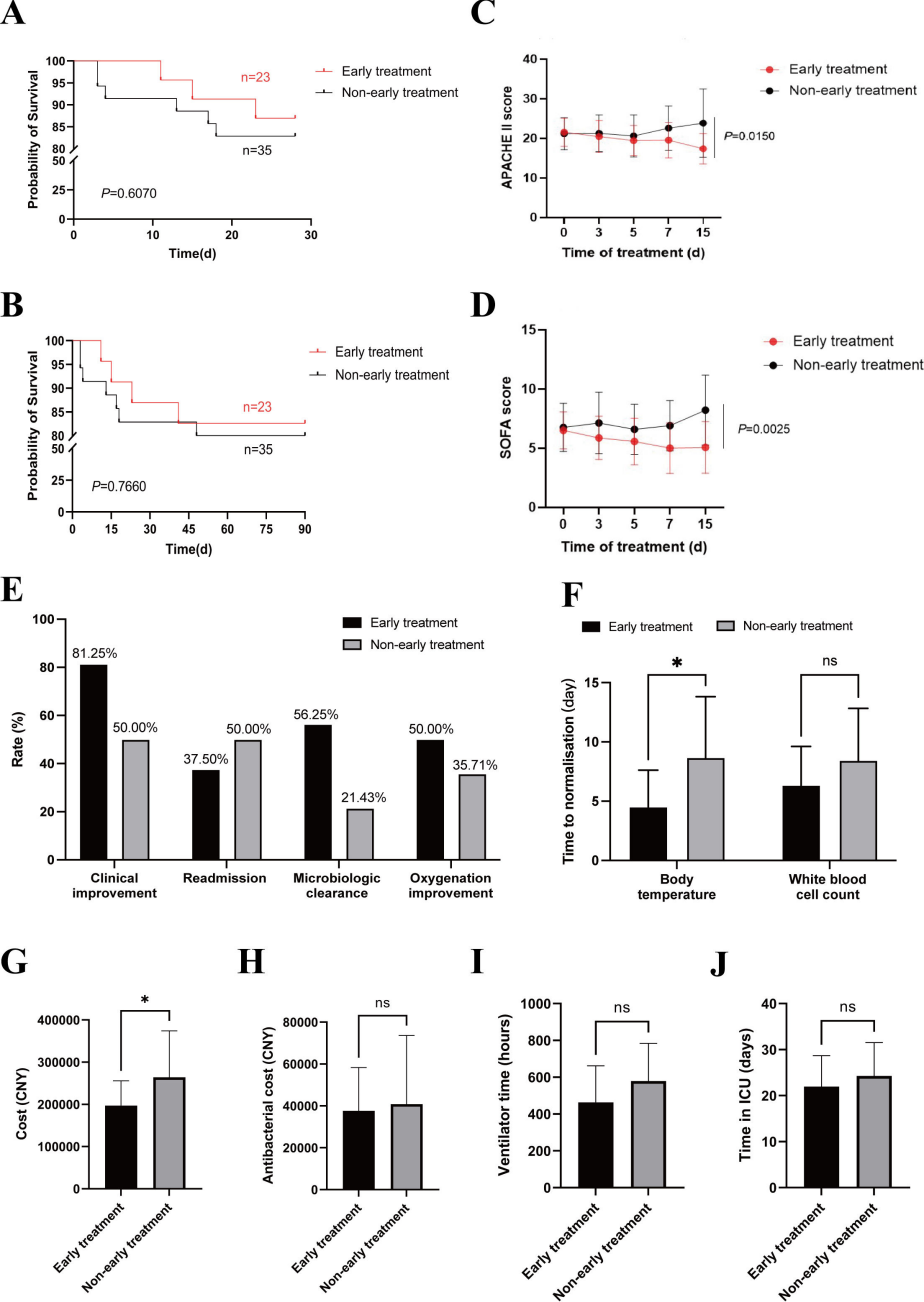
Clinical application value of Xpert Carba-R combined with LAMP to guide early antibiotic therapy. (**A and B**) Analysis of 28 and 90-day survival curves of patients in the early treatment group and the non-early treatment group. (**C and D**) Trends of APACHE II and SOFA scores in two groups within 15 days of antimicrobial treatment. (**E**) Compare the differences between the two groups in clinical improvement, readmission rate, microbial clearance rate, and oxygenation improvement. (**F**) The time for the two groups to normalize temperature and white blood cell count. Analysis of 30-day hospitalization cost (**G**), 15-day antibiotic treatment cost (**H**), 30-day ventilator use time (**I**), and 30-day ICU stay time (**J**) in the two groups. *P < 0.05; ns, not significant.

Due to the limited sample size in this study, we were unable to achieve statistical power to detect differences in the 28 and 90-day all-cause mortality rates between the two groups (Fig. 3A and B). In addition, we monitored the trend of disease severity by tracking APACHE II and SOFA scores of patients in both groups for 15 days. Due to the different initial disease severity of patients, we classified patients with APACHE II score ≥15 or SOFA score ≥4 as severe. The two scores of severe patients were statistically analyzed. We found that the APACHE II and SOFA scores in the early treatment group showed a steady downward trend over time (Fig. 3C and D). After 15 days of treatment, the early treatment group had significantly lower APACHE II (*P* = 0.015) (Fig. 3C) and SOFA scores (*P* = 0.0025) compared to the non-early treatment group (Fig. 3D). Further focusing on more detailed prognostic indicators for critically ill patients, the results showed that the early treatment group outperformed the non-early treatment group in clinical improvement, readmission rate, microbial clearance rate, and oxygenation improvement (Fig. 3E). Notably, compared with the non-early treatment group, the early treatment group achieved a clinical improvement rate of 81.25% and a microbiological eradication rate of 56.25%, showing a clear advantage. Additionally, the time to recovery of normal temperature in the early treatment group was (4.500 ± 3.119) days, compared to (8.667 ± 5.211) days in the non-early treatment group, with a statistically significant difference (*P* = 0.288). No statistically significant difference was observed between the two groups in the time to recovery of normal white blood cell count (Fig. 3F). Further comparison of the 30-day hospitalization costs of severe patients between the two groups showed that the early treatment group was lower than the non-early treatment group (*P* = 0.0427) (Fig. 3G). However, there were no significant differences in the 15-day antibiotic treatment cost (Fig. 3H), 30-day ventilator use time (Fig. 3I), and 30-day ICU stay time (Fig. 3J).

## DISCUSSION

The global prevalence of CR-GNB remains alarmingly high, posing a major threat to global health security. Infections caused by carbapenemase-producing Gram-negative bacilli are associated with high in-hospital mortality rates, reaching up to 70% (17). Early diagnosis of pneumonia caused by these resistant bacteria enables timely and targeted antibiotic therapy, which is crucial for controlling resistance rates and reducing healthcare costs. This study presents a combined diagnostic method using Xpert Carba-R and LAMP, providing a simple, time-efficient, and highly applicable approach for the early detection of carbapenemase-producing CR-GNB infections.

There were differences in the detection efficacy of the combined method across different bacterial species. Specifically, the detection efficacy for carbapenemase resistance genes in *Klebsiella pneumoniae* was the highest, while the efficacy was suboptimal in *Acinetobacter baumannii* and *Pseudomonas aeruginosa*. We think this is primarily due to the differences in the carbapenem resistance mechanisms among these bacterial species. The production of carbapenemases is the primary mechanism underlying carbapenem resistance in *Klebsiella pneumoniae*. In the United States, 92% of CRKP produce carbapenemases (18). In our study, *Klebsiella pneumoniae* predominantly produced KPC enzymes, consistent with reports that KPC is the most common carbapenemase in CRKP in China (19). This may explain the good diagnostic value of the Xpert Carba-R and LAMP combined method for the carbapenemase resistance gene of *Klebsiella pneumoniae*. In *Pseudomonas aeruginosa*, resistance is mainly mediated by efflux pump upregulation and porin loss rather than carbapenemase production (20). Among 807 CRPA isolates from 17 centers across 12 countries (2019–2021), only 33% tested carbapenemase-positive via the modified carbapenem inactivation method CRPA (21). Carbapenem resistance in *Acinetobacter baumannii* results from the coexistence of multiple mechanisms, among which the most common is the production of carbapenemases (22). Previous studies (23) have reported that class A, B, and D carbapenemase genes can all be detected in *Acinetobacter baumannii*. In our study, KPC and NDM were the major carbapenemases identified, while class D carbapenemases were not detected. However, other reports have shown that the OXA-23 genotype is predominant in *Acinetobacter baumannii* isolates from certain regions of China (24). This genotype cannot be detected by the Xpert Carba-R assay, thereby reducing the effectiveness of the combined method for identifying carbapenemase genotypes in *Acinetobacter baumannii*.

The overall sensitivity of the combined rapid detection approach was 64.87%. We further analyzed multiple factors that may have contributed to the limited sensitivity. Among specimens that were positive by conventional bacterial culture and AST but negative by the combined rapid assay, 73% (19/26) yielded *Pseudomonas aeruginosa* or *Acinetobacter baumannii* by LAMP but were negative by the Xpert Carba-R assay. This finding suggests that the Xpert Carba-R assay has limited ability to detect carbapenemase genes in *Acinetobacter baumannii* and *Pseudomonas aeruginosa* compared with *Klebsiella pneumoniae*. Such limitations may arise from differences in primer-probe efficiency, detection thresholds, or algorithmic parameters among species, which merit further optimization. In addition, the Xpert Carba-R assay targets five common carbapenemase genotypes and cannot detect resistance mechanisms unrelated to carbapenemase production or strains carrying rare carbapenemase genes. Additionally, since conventional bacterial culture and AST remain the gold standard for guiding clinical antimicrobial therapy, and our combined detection method aims to provide early guidance for targeted therapy, this study used treatment guided by bacterial culture and AST as a control to highlight its clinical value. However, bacterial culture and AST can confirm carbapenem resistance but cannot differentiate whether the mechanism involves the production of carbapenemase. The limitations of this method may result in an increased risk of false-negative rates. Theoretically, the true sensitivity of the combined assay is likely higher than the value observed in our study, a hypothesis that should be further validated through microbial sequencing analysis. From a specimen-type perspective, our study attempted to apply this combined detection directly to respiratory specimens—a broadened application that may require further optimization regarding the bacterial load threshold for such specimens. Future studies should compare this approach with established rapid carbapenemase assays to further validate its diagnostic performance and enhance its clinical applicability. Stratifying respiratory specimens into sputum and BALF samples revealed improved diagnostic performance in BALF specimens, with a sensitivity of 66.10% and an AUC of 0.814. This may be due to BALF being directly collected from the site of infection, which increases the likelihood of isolating pathogens while reducing contamination by oropharyngeal flora, thereby enhancing the specificity and sensitivity for diagnosing lower respiratory tract infections.

Among CR-GNB isolates, CRAB, CRPA, and CRKP are the most frequently identified species (25). Similarly, these three pathogens were the predominant isolates in our study. According to reports on the molecular epidemiology of carbapenemase production in China, KPC and NDM are the most commonly detected carbapenemases (26), which aligns with the findings of our study. KPC was initially isolated from *Klebsiella pneumoniae* but has since been detected in other GNB, such as *Pseudomonas aeruginosa* and *Acinetobacter baumannii* (27). In our study, KPC was primarily identified in *Acinetobacter baumannii* and *Klebsiella pneumoniae*. Currently, most reports of NDM-producing Acinetobacter species originate from China and the Middle East (28), consistent with the findings of our study, where NDM was primarily detected in *Acinetobacter baumannii* and *Pseudomonas aeruginosa*. Although OXA-48-like genes were not detected, this family—including OXA-48 and dozens of its variants, such as OXA-181 and OXA-232—shares high sequence homology and similar hydrolytic activity against carbapenems (29). The OXA-48 probe in the Xpert Carba-R assay targets a highly conserved region, allowing detection of most OXA-48 variants. While our combined assay cannot distinguish specific variants within the OXA-48 family, this limitation has minimal impact on early infection control and therapeutic decisions. Notably, the Xpert Carba-R NxG assay has expanded coverage to include Guiana extended-spectrum β-lactamase, São Paulo metallo-β-lactamase, and certain imipenem-hydrolyzing β-lactamase subtypes (30), which could further enhance detection when combined with LAMP in future studies. Recent studies have also explored the modification of LAMP for carbapenemase gene detection (31). Wu et al. (32) developed a microfluidic chip-based LAMP assay for multiplex detection of carbapenemase genes in bacterial pathogens isolated from the blood samples of patients with sepsis. Similarly, Mercado et al. (33) optimized a LAMP-based method to identify carbapenemase genes (OXA-23-like, OXA-24-like, OXA-51-like, OXA-58-like, IMP, and VIM) in *Acinetobacter baumannii* isolates from intensive care unit environments. Collectively, these findings suggest that LAMP can be modified—through the design and incorporation of primers targeting carbapenemase genes—to detect multiple carbapenemase genotypes. However, no existing LAMP assay is currently capable of simultaneously detecting both bacterial species and carbapenemase genes. Technical challenges such as complex multiplex primer design, gene polymorphism, and signal differentiation remain to be addressed.

Recent advancements in carbapenem resistance detection have focused on technologies, such as multiplex nucleic acid amplification, mass spectrometry, and high-throughput sequencing (34). These methods enable rapid and accurate detection of bacterial resistance, contributing to the growth of precision medicine in managing infectious diseases. However, high costs and the need for specialized technical expertise often limit their widespread clinical use. In contrast, molecular diagnostic techniques based on multiplex nucleic acid amplification, such as the BioFire FilmArray Pneumonia Panel and Unyvero LRT Panel, have been rapidly adopted (35). These commercially available kits can simultaneously detect common carbapenemase genotypes and major pathogenic bacteria. A key limitation of these kits is their inability to link antibiotic resistance genes with specific pathogens when multiple resistant pathogens are present in a single sample—a limitation inherent to PCR-based methods (36). Therefore, our objective is to propose a method that integrates Xpert Carba-R for carbapenemase genotype detection with LAMP for bacterial species identification, creating a genotype-pathogen match that can better guide early, targeted antibiotic therapy. The challenge in matching bacterial species to genotypes lies in the simultaneous detection of multiple bacterial species. In our study, LAMP detected a single GNB in 84% of samples, two species in 11%, and three species in 5%. Among Xpert Carba-R positive samples, LAMP detected a single GNB in 74%, two species in 15%, and three species in 11%. These results indicate that LAMP most frequently detects a single GNB. For samples where LAMP detected more than one GNB, we matched the LAMP-detected species with Xpert Carba-R results based on bacterial culture and AST. After a comprehensive review of the published literature on diagnostic platforms, such as BioFire FilmArray and Curetis Unyvero, we found that most available studies did not provide detailed diagnostic performance data or clinical impact analyses specifically for CR-GNB detection. A multicenter randomized controlled trial (37) evaluated the impact of the BioFire FilmArray Pneumonia Panel for pathogen detection in respiratory specimens on antimicrobial stewardship and clinical outcomes among patients with HAP and VAP. The study demonstrated that use of the panel significantly improved antimicrobial stewardship, with an absolute improvement of approximately 21%, but it did not establish non-inferiority for 14-day clinical cure of pneumonia. A key limitation of the study is that it was conducted exclusively in England, where the prevalence of antimicrobial resistance is relatively low; therefore, the reported data do not provide evidence regarding the diagnostic or clinical utility of this assay for CR-GNB pneumonia. Jamal et al. (38) evaluated the performance of the Curetis Unyvero system in detecting bacterial pathogens and antimicrobial-resistance determinants (including five major carbapenemase genotypes) in respiratory specimens, as well as its impact on the management of severe HAP. Among 38 infected patients, the assay identified 13 distinct resistance genes, of which the OXA-51-like carbapenemase accounted for 28.6%, and Cefotaximase-Munich β-lactamase for 12.3%; none of the five major carbapenemase genotypes were detected. Of the 15 patients with severe pneumonia, 13 (86.7%) achieved clinical and microbiological improvement. Given the relatively small patient cohort, further studies with larger sample sizes are required to validate the assay’s ability to detect CR-GNB and to determine its overall impact on clinical management.

We further evaluated the clinical prognostic value of combined detection for patients with CR-GNB pneumonia. In this study, the number of patients in the prognosis section was relatively limited, which restricted the statistical power of some prognostic indicators. In the future, it is necessary to increase the sample size of the prognosis research to further enhance the persuasiveness of the prognosis results. The APACHE II score and SOFA score are commonly used in clinical practice to evaluate the severity of diseases, and they are closely related to the prognosis of patients. Compared with the non-early treatment group, the APACHE II and SOFA scores of the early treatment group continued to decline. In the early treatment group, the APACHE II and SOFA scores began to improve within 3 days of treatment. By the 15th day, these scores were significantly lower than those in the non-early treatment group, which meant that the combined test results had a positive impact on clinical medication guidance. Based on the data of the early treatment group in terms of clinical improvement, microbial clearance rate, re-admission rate, oxygenation improvement, and fever reduction time, it suggests that this combined detection has a good potential for guiding treatment in cases of drug-resistant pneumonia. The total 30-day hospitalization cost in the early treatment group was significantly lower than that in the non-early treatment group, indicating that this combined test can effectively reduce the hospitalization expenses for patients. These results indicate that the combined detection method has a positive impact on guiding the clinical use of antibiotics, promoting the early control of CR-GNB pulmonary infection, providing greater treatment opportunities for patients with severe pneumonia, and reducing treatment costs. However, its long-term impact requires further study.

This study has certain limitations. First, since it is a single-center study with a small sample size, and all patients were from Tongji Hospital, some of the findings may not be generalizable to other settings. Further studies with larger and more diverse data sets are needed. Second, the Xpert Carba-R assay can only detect five major carbapenemase genes, and other genes that may improve diagnostic accuracy were not tested. Third, in this study, due to the limitations in detecting drug-resistant strains, we mainly evaluated the application of this combined detection method for *Klebsiella pneumoniae*. In the future, we need to further assess the detection efficacy of this combined method for other Gram-negative Enterobacterales. Fourth, *Klebsiella pneumoniae* exhibits strain heterogeneity, including differences in capsular polysaccharide types (K types) and the presence of hypervirulent (hvKP) and classical (cKP) lineages (39). These variations may influence disease severity, treatment response, and ultimately patient outcomes. While our combined detection approach provides rapid information for antibiotic selection and infection control, it is currently limited in distinguishing hvKP, as it cannot identify virulence-associated genes or capsular K-type markers. Cai et al. (40) developed a LAMP assay using an OTG visual dye system that targets the phoE gene for *Klebsiella pneumoniae* identification and two key virulence-associated genes (iroB and iucA), enabling rapid detection of hvKP. However, their LAMP design performs single-gene amplification for iroB and iucA separately, which may increase turnaround time when multiple markers are tested. In future work, optimization of multiplex LAMP strategies could allow integration of hvKP virulence genes (iucA, iroB, rmpA, rmpA2, peg-344, etc.) and capsular locus-specific sequences (cps/locus) into our combined assay, thereby enabling simultaneous screening for capsular K types and differentiation between hvKP and cKP lineages. Lastly, the lack of PCR sequencing of isolated strains limits more in-depth analysis.

In summary, the Xpert Carba-R combined with LAMP represents a promising rapid diagnostic tool for early detection of CR-GNB in respiratory specimens. Early application in high antimicrobial resistance risk patients with severe pneumonia may facilitate timely, targeted antimicrobial therapy and improve outcomes. However, results should be interpreted in the context of each patient’s resistance risk factors, clinical findings, and imaging results to avoid unnecessary treatment of colonizing organisms. Clinicians should continue to consider bacterial culture and AST, as a negative combined assay result cannot fully exclude CR-GNB infection. Rare or novel carbapenemase genotypes may escape detection, and culture-based methods remain essential for identifying non-carbapenemase-producing CR-GNB. Microbiology laboratories should ensure prompt reporting and include information on local respiratory carbapenem-resistant pathogen distribution and assay limitations to minimize misinterpretation and optimize clinical decision-making.
